# Viral proteins expressed in the protozoan parasite *Eimeria tenella* are detected by the chicken immune system

**DOI:** 10.1186/s13071-016-1756-2

**Published:** 2016-08-23

**Authors:** Virginia Marugan-Hernandez, Charlotte Cockle, Sarah Macdonald, Elaine Pegg, Colin Crouch, Damer P. Blake, Fiona M. Tomley

**Affiliations:** 1 The Royal Veterinary College, University of London, Hawkshead Lane, North Mymms, AL9 7TA UK; 2 MSD Animal Health, Walton Manor, Milton Keynes, MK7 7AJ UK

**Keywords:** *Eimeria*, Transgenic, Delivery vector, Multivalent vaccine, Poultry diseases

## Abstract

**Background:**

*Eimeria* species are parasitic protozoa that cause coccidiosis, an intestinal disease commonly characterised by malabsorption, diarrhoea and haemorrhage that is particularly important in chickens. Vaccination against chicken coccidiosis is effective using wild-type or attenuated live parasite lines. The development of protocols to express foreign proteins in *Eimeria* species has opened up the possibility of using *Eimeria* live vaccines to deliver heterologous antigens and function as multivalent vaccine vectors that could protect chickens against a range of pathogens.

**Results:**

In this study, genetic complementation was used to express immunoprotective virus antigens in *Eimeria tenella*. Infectious bursal disease virus (IBDV) causes Gumboro, an immunosuppressive disease that affects productivity and can interfere with the efficacy of poultry vaccination programmes. Infectious laryngotracheitis virus (ILTV) causes a highly transmissible respiratory disease for which strong cellular immunity and antibody responses are required for effective vaccination. Genes encoding the VP2 protein from a very virulent strain of IBDV (*vvVP2*) and glycoprotein I from ILTV (*gI*) were cloned downstream of 5’*Et-Actin* or 5’*Et-TIF* promoter regions in plasmids that also contained a mCitrine fluorescent reporter cassette under control of the 5’*Et-MIC1* promoter. The plasmids were introduced by nucleofection into *E. tenella* sporozoites, which were then used to infect chickens. Progeny oocysts were sorted by FACS and passaged several times *in vivo* until the proportion of fluorescent parasites in each transgenic population reached ~20 % and the number of transgene copies per parasite genome decreased to < 10. All populations were found to transcribe and express the transgene and induced the generation of low titre, transgene-specific antibodies when used to immunise chickens.

**Conclusions:**

*E. tenella* can express antigens of other poultry pathogens that are successfully recognised by the chicken immune system. Nonetheless, further work has to be done in order to improve the levels of expression for its future use as a multivalent vaccine vector.

**Electronic supplementary material:**

The online version of this article (doi:10.1186/s13071-016-1756-2) contains supplementary material, which is available to authorized users.

## Background

The poultry industry provides the principal source of animal protein in many developed and developing countries [1]. The adaptability of poultry production systems has fuelled expansion of the industry to most areas of the world with high productivity and relatively low costs. However, intensive systems and backyard flocks remain threatened by viral, bacterial and parasitic infections that compromise the economics of meat and egg production, cause animal welfare problems [2] and can be a significant source of zoonotic transmission to humans [3, 4]. Control of infectious diseases that challenge poultry production is critical [5] and chickens are more intensively vaccinated than any other livestock animals. In Europe, programmes include a minimum of four vaccinations for broilers whilst layers and breeders can receive up to 20 vaccinations, many to immunise against common viral diseases including Marek’s disease, Newcastle disease, Gumboro (infectious bursal disease), infectious bronchitis and infectious laryngotracheitis [6].

Coccidiosis is a disease caused by *Eimeria* species parasites. In chickens infection can incur a range of clinical symptoms including malabsorption, diarrhoea and haemorrhage. The parasites are transmitted by the faecal-oral route [7]. Coccidiosis control is predominantly achieved by chemoprophylaxis with ionophorous antibiotics and/or chemical anticoccidials. Vaccination with formulations of live wild-type or attenuated parasites is widely used, and very effective, in layers and breeders and with an increased role in the broiler market due to new regulations (e.g. Veterinary Feed Directive in the US) and a preference of retailers to sell meat without antibiotics or with reduced antibiotics. [8]. Protocols which allow genetic complementation of *Eimeria* to express heterologous protein-coding sequences [9] raises the interesting possibility that existing *Eimeria* vaccines could be engineered to express antigens derived from a wide range of poultry pathogens [10, 11]. *Eimeria* genomes are much larger than those of viral vectors, in consequence they can tolerate the insertion and expression of several foreign antigens. They induce a broad range of potent immune responses following oral administration [12] having the potential to be exploited as a flexible oral vaccine vector for intracellular and extracellular pathogens.

In this study we have expressed in *Eimeria tenella* specific antigens that have already been proven capable of inducing protection against two economically important viral diseases of chickens. Infectious bursal disease virus (IBDV) is a birnavirus that causes Gumboro, an acute and highly contagious disease that destroys B lymphocytes within the bursa of Fabricius, causing morbidity, mortality and immunosuppression, leaving birds susceptible to other infections and compromising vaccination programmes [13]. The IBDV major capsid protein VP2 is the target of antiviral neutralising antibodies [14–16] and is already used in a licenced HVT-vectored vaccine (Vaxxitec, Merial). Infectious laryngotracheitis virus (ILTV) is an alphaherpesvirus that causes highly contagious acute respiratory disease leading to growth depression, reduced egg production and death [17]. A fowlpox virus-vectored vaccine that expresses ILTV membrane glycoprotein B (gB) and DNA packaging protein UL-32 is available (Ceva Bioimmune Vectormune® FP-LT). Additionally, the ILTV membrane glycoproteins gD and gI have been expressed in the HVT-vectored vaccine Innovax®-ILT (MSD Animal Heath).

Therefore, VP2 from a very virulent strain of IBDV (termed vvVP2) and gI from ILTV were each expressed in populations of transgenic *E. tenella* parasites under the control of *E. tenella* promoter sequences 5*’Et-Actin* [9] or 5*’Et-TIF* (a genomic region upstream of gene ETH_00025365 encoding a putative translation initiation factor, E. Pegg, unpublished) in order to test the suitability of the parasite as an oral vaccine vehicle. The different transgenic populations were used to immunise chickens and their ability to develop any type of immune response was analysed.

## Methods

### Parasites and birds

The *E. tenella* Wisconsin strain was used throughout [18]. Oocyst purification, excystation and sporozoite purification were performed as described previously [19, 20]. Parasites were propagated in 3 week-old White Leghorn chickens reared under specific pathogen free conditions and dosed with 4 × 10^3^ sporulated oocysts (by oral intubation), or with 0.75 × 10^6^ transfected sporozoites (by cloacal drinking [9]). Transfected sporozoites were also used to infect monolayers of Madin-Darby bovine kidney (MDBK; ECACC-Sigma-Aldrich, Salisbury, UK) cells as previously described [21].

### Polymerase Chain Reaction (PCR) and molecular cloning

PCR amplification was performed using Taq DNA Polymerase (Invitrogen, California, USA). Primers were designed using CLC Main Workbench software (CLC bio, Aarhus, Denmark) and synthesised by Sigma. Each PCR reaction contained DNA template, forward and reverse primers (20 pmol), *Taq* polymerase (0.5 U), dNTPs (0.2 mM; Promega, Hampshire, UK), Tris-HCl (20 mM), KCl (50 mM) and MgCl_2_ (2 mM). Standard cycle parameters were 1 × (5 min at 95 ^o^C), 30 × (30 s at 94 ^o^C, 30 s at 56 ^o^C and 1 min at 72 ^o^C) and 1 × (10 min at 72 ^o^C). Post-amplification PCR products were resolved by agarose gel electrophoresis, excised and purified using MinElute Gel Extraction (Qiagen, West Sussex, UK) and cloned into pGEM®-T Easy (Promega). Plasmids were propagated in *Escherichia coli* XL1-Blue (Stratagene, California, USA), purified using QIAprep Spin Miniprep (Qiagen) and sequenced (GATC Biotech, Konstanz, Germany) as described by the respective manufacturers. Sequence analysis was done using CLC Main Workbench.

### Plasmid constructs for transgene expression in *E. tenella*

**Fig. 1:**
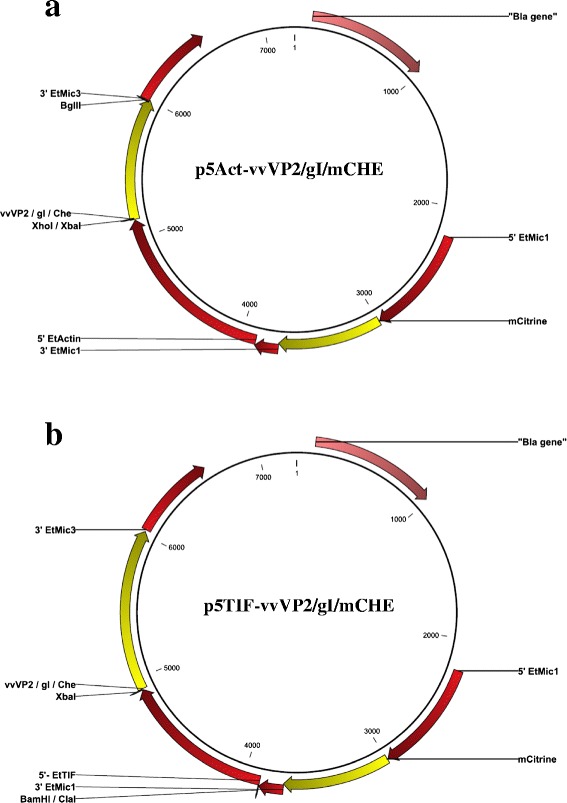
Transfection constructs used for the genetic complementation of *E. tenella* with the viral genes *vvVP2* (IBDV), *gI* (ILTV) or the reporter gene *mCherry* under the control of **a** the 5*’Et-Actin* promoter region or **b** the 5’*Et-TIF* promoter region. The positions of restriction sites used for cloning the gene or promoter region are indicated for each plasmid

Starting plasmid pCIT_CjaA [10] contained the *mCitrine* coding sequence directly downstream of the 5’*Et-Mic1* promoter, and a second expression cassette where the viral genes of interest could be cloned downstream of either the 5’*Et-Actin* or 5’*Et-TIF* promoter region (Fig. 1).

To generate plasmids using the 5’*Et-Actin* promoter (Table 1, Fig. 1a), primers incorporating *Xho* I (for *vvVP2* and *gI*) or *Xba* I (for *mCherry*) and *Bgl* II restriction sites (Additional file 1: Table S1) were used to amplify the coding sequences of IBDV *vvVP2* (accession number HG974565.1; derived from F52/70 Faragher challenge virus, GDV-232/270890) and ILTV *gI* (accession number KP677883.1; derived from Innovax-ILT vaccine, HVT-138), combined in the plasmid pVEC211 (kindly provided by MSD Animal Health) and *mCherry* fluorescent reporter (from Core Construct 1; [9]). The PCR products were cloned into pGEM®-T Easy (Promega) and digested with *Xho* I or *Xba* I and *Bgl* II (NEB, Hertfordshire, UK) for subsequent ligation into the pCIT_CjaA backbone, digested in parallel using the same endonucleases. For the second set of plasmids (Table 1, Fig. 1b), the 5’*Et-Actin* promoter sequence was replaced by the ETH_00025365 (putative translation initiation factor - TIF) promoter region, *5’Et-TIF*. A region of ~1 kb upstream of the ETH_00025365 coding sequence was amplified using primers including *Cla* I (for *vvVP2*) or *Bam* HI (for *gI* and *mCherry*) and *Xba* I restriction sites (Additional file 1: Table S1). The PCR products were cloned into pGEM®-T Easy (Promega) and digested by *Cla* I or *Bam* HI and *Xba* I (NEB) for subsequent ligation into the corresponding plasmid. The correct sequence and insertions for each amplified product were confirmed by sequencing (GATC Biotech). Final plasmid DNAs were amplified in *E. coli* XL1-Blue (Stratagene), purified using an EndoFree Plasmid Maxi kit (Qiagen), linearized by digestion with *Psi* I (0.5 U per 1 μg of plasmid DNA, NEB, confirmed by running out in an 0.8 % agarose gel), precipitated in ethanol-sodium acetate and quantified by NanoDrop (Thermo, Massachusetts, USA) as recommended by the respective manufacturers.

**Table 1:** Summary of plasmid constructs used for genetic complementation of *E. tenella*

Plasmid	Fluorescent reporter	Target gene	Gene promoter
p5Act-vvVP2^a^	5’*EtMic1*-*mCitrine*-3’*EtMic1*	*vvVP2* (IBDV)	5’*Et-Act*
p5Act-gI^a^	5’*EtMic1*-*mCitrine*-3’*EtMic1*	*gI* (ILTV)	5’*Et-Act*
p5Act-mChe^b^	5’*EtMic1*-*mCitrine*-3’*EtMic1*	*mCherry*	5’*Et-Act*
p5TIF-vvVP2^a^	5’*EtMic1*-*mCitrine*-3’*EtMic1*	*vvVP2* (IBDV)	5’*Et-TIF*
p5TIF-gI^a^	5’*EtMic1*-*mCitrine*-3’*EtMic1*	*gI* (ILTV)	5’*Et-TIF*
p5TIF-mChe^b^	5’*EtMic1*-*mCitrine*-3’*EtMic1*	*mCherry*	5’*Et-TIF*

^a^Used for the generation of stable populations

^b^Used to test mCherry expression in transient transfection

### Transfection of *E. tenella* sporozoites

Transfection of purified sporozoites was carried out in 16-well strips using programme EO114 of a Nucleofector 4D (Lonza, Basel, Switzerland). Restriction enzyme-mediated integration (REMI) was applied to increase transfection efficiency [9, 22]. For transfection, four wells were used, each containing 1 × 10^6^ sporozoites in 20 μl P3 buffer (Lonza), 12 μg of plasmid DNA (in 2 μl) and 6 U *Psi* I. Each well was then shocked and 80 μl of RPMI medium (Sigma, Suffolk, UK) was added per well and incubated at room temperature for 20 min. Transfected sporozoites from the four wells were combined and used to infect MDBK cell cultures and/or to infect chickens.

Sporozoite survival rates were estimated after transfection using Trypan blue dye exclusion (Invitrogen). One million of the initial transfected sporozoites were incubated with MDBK cells at 41 °C - 5 % CO_2_ in 24 well plates, washed with Advanced DMEM (Gibco, Leicestershire, UK) (supplemented with 2 % of foetal bovine serum (FBS; Sigma) and 100 U of Penicillin/Streptomycin (Fisher, Leicestershire, UK)) after 2 h, and after 24 h the presence of parasites expressing mCitrine and/or mCherry was confirmed by fluorescent microscopy (Leica DMI3000B - DCF365FX, Wetzlar, Germany).

### Obtaining populations of transgenic oocysts

Chickens were infected with transfected sporozoites equivalent to 0.75 × 10^6^ of the starting sporozoite sample by direct cloacal inoculation [9]. The precise live sporozoite dose was calculated subsequently based upon survival during transfection. After 1 week, oocysts were harvested from caecal contents and sporulated by incubation and agitation in potassium dichromate (2 % w/v) for 3 days at 27 °C. The proportion of fluorescent parasites in each oocyst population was determined by microscope counts (Leica DMI3000B – DCF365FX) and fluorescence-activated cell sorting (FACS) on a BD FACS Aria^TM^ III (BD, California, USA). Further passages of fluorescent-sorted sporulated oocysts through chickens were carried out to increase the proportion of transgenic parasites within the population.

### Flow-activated cell sorting (FACS) of transgenic populations

Expression of *mCitrine* was used to identify parasites that were successfully transfected with test plasmids. Fluorescent oocysts were sorted in the BD FACS Aria™ III (BD) using an excitation wavelength of 488 nm, emission filter of 330/30, a 100 micron nozzle and pressure of 20 psi. Sample purity (fluorescent events/total events) was recorded for each population before and after sorting.

### Nucleic acid and protein isolation and complementary DNA synthesis

TRIzol® Regent (Invitrogen) was used for simultaneous isolation of RNA, genomic DNA (gDNA) and protein from oocysts. Two million sporulated oocysts were used and extractions were performed following the manufacturer’s recommendations. RNA and protein pellets were stored at -80 °C, gDNA at -20 °C, until further use. Complementary DNA (cDNA) was synthesized from total RNA using SuperScript II® reverse transcriptase (200 U; Invitrogen), random hexamer primers (2.5 μM; Applied Biosystems, Leicestershire, UK) and RNaseOUT™ recombinant ribonuclease inhibitor (40U; Invitrogen) according to the manufacturer’s instructions. cDNA was stored at -20 °C until further use. To confirm the absence of gDNA in the cDNA samples, primers for the *Et-Actin* gene were designed across an intron (Additional file 1: Table S1) such that gDNA contamination of cDNA would result in amplification of two fragments. The presence of transgene and transgene transcription was analysed for each oocyst population by PCR of gDNA and cDNA samples with specific primers (Additional file 1: Table S1).

### Real time quantitative PCR

Real time quantitative PCR (qPCR) was performed in a CFX96 Touch® Real-Time PCR Detection System (Bio-Rad, Hertfordshire, UK) using DNA-binding dye SsoFastTM EvaGreen® Supermix (Bio-Rad). The PCR mixture contained 1 μl DNA template (gDNA or cDNA), SsoFast EvaGreen supermix (10 μl), forward and reverse primers (250 nM) and RNase/DNase-free water in a final volume of 20 μl. Amplification was performed according to the manufacturer’s protocol, with one cycle at 95 °C for 1 min, followed by 40 cycles at 95 °C for 15 s and 60 °C for 30 s. All samples were processed in triplicate, supplemented by a positive control dilution series and a no template negative control. Data were analysed with the Bio-Rad CFX Manager software (Bio-Rad).

The average copy number of transgenes per parasite genome was determined for each oocyst population using gDNA and specific primers for *mCitrine* (to estimate the transgene copy number) and *Eimeria* spp. 5S rDNA (to estimate the total number of genomes) (Additional file 1: Table S1); as described previously [9].

Transgene transcription was quantified from cDNA using specific primers (Additional file 1: Table S1) and compared with serial dilutions of DNA standard templates for each transgene. pGEM®-T Easy (Promega) plasmids containing the *vvVP2*, *gI* or *mCitrine* coding sequences were used as the DNA standard templates. Standard curves were prepared for all genes from 10^8^ to 10^2^ plasmid copies, against which transcript copy numbers were quantified. Data were analysed using the Kruskal-Wallis test followed by Mann-Whitney test for pairwise comparison (GraphPad Prism 6 v.6.00, California, USA).

### Expression of rec-vvVP2, rec-gI and rec-gD recombinant proteins

Sequences corresponding to immunogenic regions (predicted by CLC Main Workbench software; CLC bio) of IBDV vvVP2 (nt199-nt657; accession number HG974565.1) and ILTV gI (nt420-nt810; KP677883.1) and ILTV gD (nt645-nt1182; KP677882.1) were amplified and cloned into plasmid vector pET32b (+) (Novagen, Hertfordshire, UK). DNA fragments were amplified from plasmid pVEC211 (kindly provided from MSD Animal Health) incorporating *Hind* III and *Xho* I restriction sites using the primers listed in Additional file 1: Table S1. PCR products were sub-cloned into pGEM®-T Easy (Promega) and digested by *Hind* III and *Xho* I (NEB) for ligation into pET32b(+), digested in parallel using the same endonucleases.

Recombinant proteins were expressed as polyhistidine (His6) and thioredoxin fusions in BL21 (DE3) pLysS *E. coli* (Stratagene). Bacteria were lysed overnight with phosphate buffer, 40 mM imidazole, 5 % (v/v) glycerol, 0.5 % (v/v) triton x 100, lysozyme (5KU/gr; Novagen) and benzonase (25U/ml; Sigma). Insoluble recombinant proteins (rec-vvVP2 and rec-gI) were denatured in phosphate buffer containing 8 M urea and 40 mM imidazole. Recombinant proteins were purified by immobilized metal ion affinity chromatography (IMAC) using HisTrap™ HP columns following the manufacturers protocols (GE Healthcare, Buckinghamshire, UK). After elution in 500 mM imidazole, recombinant proteins were checked by SDS-PAGE (Invitrogen) and protein concentration estimated by Bradford assay (Invitrogen) using serial dilutions of bovine serum albumin (BSA) as standard.

### Immunogenicity trial

To evaluate the potential of *E. tenella* transgenic oocyst populations to stimulate specific antibody responses against an expressed transgene, groups of 21-day-old White Leghorn chickens were immunised orally with oocysts containing IBDV *vvVP2* or ILTV *gI* transgenes under the control of either the 5*’Et-Actin* or 5*’Et-TIF* promoter. Birds dosed with *E. tenella* wild type oocysts or with PBS were used as controls (Table 2). To mimic the re-cycling of parasites that occurs in natural infections, chickens were infected on more than one occasion, as stated in Table 2. Faeces were collected from each group of birds at 3 and 7 days after the first infection and oocysts were recovered to demonstrate successful inoculation of birds with transgenic or wild type *E. tenella*. Data were analysed by Chi-square test followed by Fisher’s exact test (GraphPad Prism 6 v.6.00).

**Table 2:** Experimental groups used for immunisation trial

Group	Parasite population	No. of birds	100 oocysts	500 oocysts	3000 oocysts	5000 oocysts
1	*Et-*Act-vvVP2-P6	12	day 0	day 4	day 6	day 13
2	*Et-*TIF-vvVP2-P2	12	day 0	day 4	day 6	day 13
3	*E. tenella* (wt)	6	day 0	day 4	day 6	day 13
4	*Et-*Act-gI-P5	12	day 0	day 2	day 8	day 14
5	*Et-*TIF-gI-P2	12	day 0	day 2	day 8	day 14
6	*E. tenella* (wt)	6	day 0	day 2	day 8	day 14

All birds were cardiac bled under terminal anaesthesia at the end of the study at 57 days of age (42 days after the last immunisation). Bloods were incubated at 37 °C for 2 h, then transferred to 4 °C overnight and serum collected after centrifugation at 10,000 g for 10 min and stored at -20 °C until further use.

### SDS-polyacrylamide gel electrophoresis (PAGE), Western blot and ELISA

Total protein extracted by TRIzol® Reagent (Invitrogen) from passaged oocysts of transgenic or wild type parasites was electrophoresed through NuPAGE 4–12 % Bis-Tris 10 well gels (Invitrogen) in Laemmli loading buffer (Sigma). Proteins were transferred to nitrocellulose membranes (GE Healthcare) in a semidry system following the manufacturers protocols (Invitrogen) and blocked in 5 % (w/v) blotting grade non-fat milk (Bio-Rad) overnight. Membranes were incubated for 1 hour with a 1/100 dilution of a set of sera kindly provided by MSD Animal Health: anti-mouse MCA-LTV-Mab6-INT and anti-chicken ILT Spafas for gI; anti-mouse MCA 10-INT and anti-mouse IBDV R63 MoAb for vvVP2. After washing three times with TBS-Tween 0.05 % (v/v) membranes were incubated for 1 hour with goat anti-mouse IgG antibody horseradish peroxidase (HRP) conjugate (Merk Millipore, Hertfordshire, UK) or rabbit anti-chicken IgG antibody HRP conjugate (Merk Millipore). Membranes were washed three times with TBS-Tween 0.05 % (v/v), once with TBS and finally with distilled water before adding Luminata substrate (Merck Millipore). Chemiluminiscence was visualised in a G:BOX (Syngene, Cambridge, UK) and images were taken with GeneSnap 7.12 software (Syngene).

To determine whether immunised chickens developed specific antibodies against vvVP2, gI or *E. tenella*, 5 μg of rec-vvVP2 or rec-gI recombinant proteins or 10^7^*E. tenella* sporozoites were electrophoresed through NuPAGE 4–12 % Bis-Tris 1 well gels (Invitrogen) in Laemmli loading buffer (Sigma). Proteins were transferred and blocked as described above. The membranes were incubated with different dilutions of chicken serum (1/200 - 1/100 - 1/50) and corresponding controls (sera from chickens immunised with Vaxxitec for vvVP2 and with ILT Spafas for gI) in a multiscreen apparatus (Bio-Rad) for 1 hour, washed three times with TBS-Tween 0.05 % (v/v) and incubated again for 1 hour with rabbit anti-chicken IgG antibody HRP conjugate (Merck Millipore). Membranes were washed and developed as described above. Antisera were also analysed by enzyme-linked immunosorbent assay (ELISA) at The Service Laboratory (Boxmeer, Netherlands) using an IDEXX IBD Antibody Test Kit for vvVP2 and the ProFLOK® ITL Antibody Test Kit. The presence of neutralising antibodies for vvVP2 were also analysed using the GDV D78 VN assay.

### Lymphoproliferation assays

An experiment replicating the immunization schedule of the immunogenicity assay (as above and Table 2) using 7-day-old chickens, was performed to analyse the ability of vvVP2 and gI antigens, expressed by *E. tenella,* to induce lymphocyte proliferation. In this experiment groups of four chickens were used. For peripheral blood mononuclear cells (PBMC), birds were bled from the wing vein at 7 and 14 days after the last immunization; blood was treated with heparin to avoid coagulation (0.3 U/ μl) and 1 ml was mixed with Histopaque 1077 (Sigma) (1:1). For splenocytes, spleens were removed at post-mortem (21 days after the last immunization), sliced, washed with PBS, passed through a cell strainer (40 μm) and the homogenate (6 ml) was mixed with Histopaque 1077 (Sigma) (1:1). Samples were centrifuged at 1250 g for 30 min and the interphase recovered and washed with 1 ml (PBMC) or 5 ml (splenocytes) of PBS three times (600 g – 15 min), then resuspended in 1 ml or 5 ml RPMI containing 10 % (v/v) FBS. Isolated cells were counted and 10^5^ cells added per well in 96-well-plates, using 12 wells per group.

PBMC and splenocytes were stimulated with Concanavalin A (ConA; 5 μg/ml; Sigma), soluble *E. tenella* antigen (10 μg/ml), recombinant protein (vvVP2 or gI; 10 μg/ml) or PBS and incubated at 41 °C with 5 % CO_2_ for 5 days. Cell proliferation was monitored by the incorporation of tritiated thymidine (^3^H-Tdr, specific activity 485 GBq/mmol; Thermo), 37 kBq ^3^H-Tdr were added for the final 24 h of incubation. Plates were then stored at -20 °C for at least 1 day and cells were harvested onto with a semi-automated cell harvester (Skatron, California, USA) and counted in a 1205 Betaplate™ counter (Wallac, Zurich, Switzerland). Data were analysed by a one-way ANOVA with *post-hoc* Bonferroni test (SPSS Statistics 22, New York, USA).

## Results

### Transiently transfected *E. tenella* sporozoites expressed foreign antigens under the regulation of two different promoter regions

**Fig. 2:**
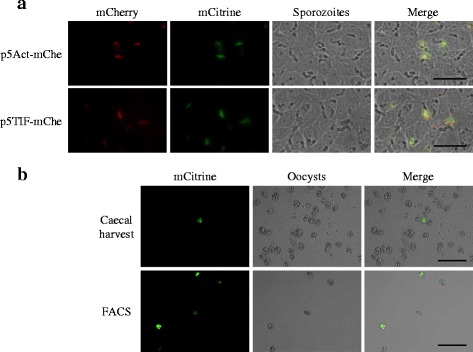
Transgenic parasites expressing fluorescent reporters. **a** Transiently transfected sporozoites expressing mCherry under the control of 5*’Et-Actin* (panel above) or 5*’Et-TIF* (panel below) and mCitrine under the control of 5*’Et-Mic1*. *Scale-bars*: 40 μm. **b** Oocysts recovered by caecal harvest following *in vivo* passage (panel above) and after enrichment by FACS (panel below). *Scale-bars*: 100 μm

The functionality of promoters 5*’Et-Actin* and 5*’Et-TIF* was confirmed by detection of red fluorescence in sporozoites transfected with constructs containing *mCherry* cloned downstream of each promoter (Table 1, Fig. 2a). Quantification of transgene copy number per parasite genome and the level of transgene transcription was not carried out in these short-term sporozoite cultures because non-integrated plasmid was also detected, evidenced by a number of *mCitrine* copies per genome determined by qPCR to exceed 35,000 (data not shown).

### Viral proteins were expressed in transgenic populations of sporulated oocysts, and transgene copy number per parasite genome reduced after *in vivo* parasite passage

**Fig. 3:**
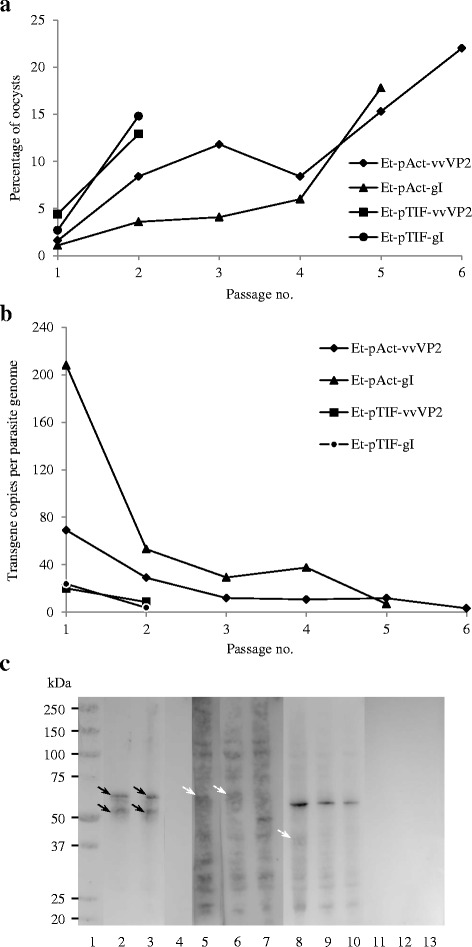
Analysis of transgenic *E. tenella* populations. **a** The percentage fluorescence detected for each transgenic population with successive *in vivo* passage. **b** Number of transgene copies per parasite genome for each transgenic population with successive passage through chickens. The number of copies was calculated using a standard curve of *E. tenella* gDNA. **c** Western blot of the different transgenic populations; *Et-*Act-gI-P5: lanes 2, 5; *Et-*TIF-gI-P2: lanes 3, 6; *Et-*Act-vvVP2-P6: lanes 8, 11; *Et-*TIF-vvVP2-P2: lanes 9, 12; and *E. tenella* (wt): lanes 4, 7, 10, 13; incubated with MCA-LTV-Mab6-INT (lanes 2–4) or ILT Spafas (lanes 5–7) for gI recognition and MCA 10-INT (lanes 8–10) or IBDV R63 MoAb (lanes 11–13) for vvVP2 recognition. Lane 1 is the Mw. Solid black arrows indicate specific band recognition with a size of ~62 kDa for gI and a processed form of it ~56 kDa (lines 2 and 3). Dotted white arrows indicate potential band recognition with sizes of ~62 kDa for gI and ~42 kDa for vvVP2 (lines 5, 6 and 8)

Sporozoites transfected with the different plasmids (Table 1) showed a survival between 17.5 and 24.0 % and were used to infect chickens and generate oocysts (Table 3). The populations that emerged contained mixtures of fluorescent and non-fluorescent oocysts. Each population was enriched for fluorescent oocysts after sporulation by FACS sorting (85–95 % purity) and re-passaged in chickens on two to six occasions per plasmid (Table 3, Fig. 2b). The proportion of fluorescent oocysts increased after passaging, but did not exceed 22 % in any example (Fig. 3a).

**Table 3:** Sporozoite survival after transfection, the numbers used to initiate the primary infection and the total number of generations produced for each plasmid

Transfected plasmid	Parasite survival (%)	Number of live sporozoites dosed per chicken	Number of *in vivo* passages
p5Act-vvVP2	17.5	131,250	6
p5Act-gI	24.0	180,000	5
p5TIF-vvVP2	21.6	162,080	2
p5TIF-gI	22.4	168,341	2

The presence and expression of *mCitrine* in transgenic parasites was evidenced by fluorescence (Fig. 2b). The presence of the second cassette containing each foreign gene was confirmed by PCR with gene-specific primers (Additional file 1: Table S1). These same primers were also used to confirm transgene transcription by real time PCR (RT-PCR).

Variation in the number of transgene copies per genome was analysed for each generation of transgenic parasites. The transgene copy number dropped dramatically after two passages, and thereafter further passages were done until they stabilised at an average of less than ten copies per genome (Fig. 3b).

To determine the occurrence of transgene expression a band of the expected size (~62 kDa) was detected by Western blotting using extracts of transgenic populations expressing gI (*Et-*Act-gI and *Et-*TIF-gI) when they were incubated with MCA-LTV-Mab6-INT (Fig. 3c). A second band of ~56 KDa was also detected in both transgenic populations but was absent from the wild-type parasites. Recognition with a second antibody, ILT Spafas, was not conclusive (Fig. 3c). In contrast no clear recognition of a protein of the expected size (~42 kDa) was observed in extracts of transgenic populations expressing vvVP2 (*Et-*Act-vvVP2 and *Et-*TIF-vvVP2) (Fig. 3c), only an unspecific band (~59 kDa) which was conserved within the wild-type sample was observed.

### Transcription levels of viral transgenes in sporulated oocysts were independent of the promoter region used

Each plasmid carried two coding sequences under the regulation of different promoters, 5’*Et-Mic1* linked to *mCitrine* and 5’*Et-Actin* or 5’*Et-TIF* linked to *vvVP2* or *gI*. In order to determine the relative level of transcription of 5’*Et-Actin* or 5’*Et-TIF* in relation to 5’*Et-Mic1* a reverse transcription qPCR was performed. The transcription of *mCitrine* linked to *5’Et-Mic1* was always higher than transcription of *vvVP2* or *gI* linked to either 5*’Et-Actin or* 5’*Et’TIF* (Table 4). When expressing *gI*, 5’*Et’TIF* lead to a stronger transcription than 5*’Et-Actin* (an average of 2.1 times more). However, when expressing *vvVP2*, the promoter region 5*’Et-Actin* led to a stronger transcription than 5’*Et’TIF* (an average 9.4 times more).

**Table 4:** Relative transcription levels of the foreign gene in cassette 2 (*vvVP2* or *gI*) compared with *mCitrine* in cassette 1 in different transgenic populations

Population	*mCitrine/vvVP2* ^c^	Population	*mCitrine/gI* ^c^
*Et*-Act-vvVP2-P1	34.7^a^	*Et*-Act-gI-P1	139.0^a^
*Et*-Act-vvVP2-P2	4.2^a^	*Et*-Act-gI -P2	36.3^a^
*Et*-Act-vvVP2-P3	4.2^a^	*Et*-Act-gI -P3	82.6^a^
*Et*-Act-vvVP2-P4	6.4^a^	*Et*-Act-gI -P4	62.8^a^
*Et*-Act-vvVP2-P5	1.7^a^	*Et*-Act-gI -P5	182.8^a^
*Et*-Act-vvVP2-P6	0.4^a^		
*Et*-TIF-vvVP2-P1	67.2^b^	*Et*-TIF-gI-P1	76.9^b^
*Et*-TIF-vvVP2-P2	94.9^b^	*Et*-TIF-gI-P2	17.4^b^

^a^Equivalent to 5’*Et-Mic1* / 5’*Et-Actin* ratio

^b^Equivalent to 5’*Et-Mic1* / 5’*Et-TIF ratio*

^c^Ratio indicates the times *mCitrine* is higher than the other transcript

### Immunisation of chickens with transgenic parasites led to the generation of specific antibodies but did not trigger lymphocyte proliferation in a re-stimulation assay

**Fig. 4:**
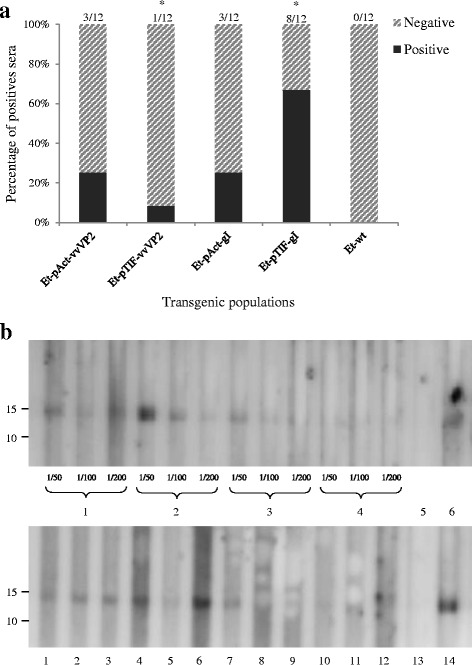
Humoral immune responses by chickens immunised with *E. tenella* transgenic populations and wild type parasites. **a** Proportion of chickens presenting a positive reaction against rec-vvVP2 or rec-gI by western blot. * Indicates significant differences between groups in the frequency of recombinant protein detection (*P* < 0.05, Fisher’s exact test). **b** Western blot of chicken sera against rec-vvVP2 (panel above); lanes 1-2: positive sera from chickens immunised with *Et-*Act-vvVP2-P6 at dilutions 1/50, 1/100 or 1/200; lanes 3-4: positive and negative sera from chickens immunised with *Et-*TIF-vvVP2-P2 at dilutions 1/50, 1/100 or 1/200; lane 5: negative control (sera from chickens immunised with *E. tenella* (wt) at dilution 1/50); lane 6: positive control (sera from chickens immunised with Vaxxitec at dilution 1/100) and Western blot of chicken sera against rec-gI (panel below); lanes 1–7: chicken reacting positive (1–3: *Et-*Act-gI-P5 at dilution 1/50; 4–7: *Et-*TIF-gI-P2 at dilution 1/50); lanes 8–12: chicken reacting negative (8–9: *Et-*Act-gI-P5 at dilution 1/50; 10–12: *Et-*TIF-gI-P2 at dilution 1/50); lane 13: negative control (sera from chickens immunised with *E. tenella* (wt) at dilution 1/50); lane 14: positive control (anti-chicken ILT Spafas at dilution 1/200). Rabbit anti-chicken IgG antibody HRP conjugate (Merk Millipore) at 1/1000 dilution was used as secondary antibody

The presence of specific antibodies 42 days after the last immunisation against rec-vvVP2 or rec-gI was tested by Western blot for all groups of chickens immunised using the relevant transgenic parasite population, with between one and eight of twelve birds found to be positive per group (Fig. 4a). However, the sera were reactive only at high concentrations (Fig. 4b), none were positive when tested with commercial ELISA kits and the ELISA specific for virus neutralising antibodies resulted in a 2Log titre of < 5 (equivalent to a titre of less than 1 in 32; Additional file 1: Table S2). The frequency of antibody detection by Western blot was significantly higher for the group immunised with *Et*-TIF-gI compared to that immunised with *Et*-TIF-vvVP2 (*P* < 0.05, Fisher’s exact test), but there were no other significant differences found between any other groups. All sera from immunised chickens, whether positive or negative against the recombinant proteins, were positive when tested against *E. tenella* extracts (Additional file 1: Figure S1).

**Fig. 5:**
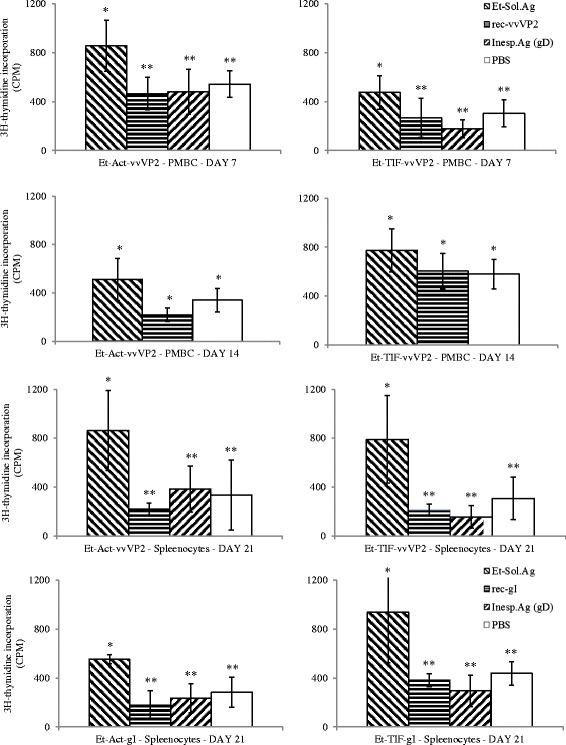
Proliferation of PMBC and splenocytes isolated from chickens immunised with the different transgenic populations (*Et*-Act-vvVP2, *Et*-TIF-vvVP2, *Et*-Act-gI or *Et*-TIF-gI) after stimulation with *E. tenella* soluble antigens, the corresponding recombinant antigen (rec-vvVP2 or rec-gI), an unspecific antigen (rec-gD) or PBS. *, ** indicate different significances, only *E. tenella* soluble extract induced linfoproliferation at days 7 (*Et*-Act-vvVP2: *F*_(3,46)_ = 11.825, *P* < 0.00001; *Et*-TIF-vvVP2: *F*_(3,45)_ = 9.041, *P* < 0.00001; one-way ANOVA) and 21 (*Et*-Act-vvVP2: *F*_(3,24)_ = 12.439, *P* < 0.00001; *Et*-TIF-vvVP2: *F*_(3,42)_ = 9.470, *P* < 0.00001; *Et*-Act-gI: *F*_(3,35)_ = 11.769, *P* < 0.00001; *Et*-TIF-gI: *F*_(3,28)_ = 11.870, *P* < 0.00001; one-way ANOVA). No significant linfoproliferation was observed at day 14 (*Et*-Act-vvVP2: *F*_(2,10)_ = 2.557, *P* = 0.127; *Et*-TIF-vvVP2: *F*_(2,27)_ = 1.091, *P* = 0.350; one-way ANOVA)

PBMC from birds immunised with transgenic parasites expressing vvVP2 (*Et-*Act-vvVP2 and *Et-*TIF-vvVP2) were isolated and re-stimulated *in vitro* at day 7 and 14 post-immunisation. Proliferation was observed when they were stimulated with the unspecific stimulator ConA (data not shown) or with *E. tenella* soluble antigen only at day 7. However, there were no significant changes (*P* > 0.05, one-way ANOVA) when rec-vvVP2 was added to the media compared with the PBS controls or a non-specific recombinant protein (rec-gD) (Fig. 5). Birds immunised with transgenic parasites expressing gI (*Et-*Act-gI and *Et-*TIF-gI) were not tested at these time points. Splenocytes from birds of all immunised groups were isolated and stimulated at day 21 post-immunisation. Similar to what was observed for the PBMC, only ConA (data not shown) and *E. tenella* soluble antigen were able to stimulate the cells (Fig. 5).

## Discussion

In this study new transgenic populations of *E. tenella* expressing antigens from either of two viruses that cause serious diseases in chickens, IBDV and ILTV, have been generated. Oral immunisation of chickens using these parasites was able to induce specific antibody responses against the viral antigens in some, but not all individuals. The idea of using *Eimeria* parasites as multivalent vaccine vectors was suggested several years ago, and to date some partial success has been reported. Clark et al. [10] expressed *Campylobacter jejuni* antigen CjaA in *E. tenella* and whilst birds immunised with the transgenic parasites were protected (~90 % reduction in *C. jejuni* in the caeca 2 weeks after challenge, compared to controls) evidence of immunological recognition of the antigen by the chicken was inconclusive. Moreover, Liu et al. [11] generated parasites expressing the M2 protein of the avian influenza virus, but found no evidence of antibody or cellular responses. The only immune responses reported in chickens raised by transgenic *Eimeria* has been against a fluorescent reporter protein (EYFP) [23], therefore, this is the first time that antibodies against viral proteins expressed in transgenic *Eimeria* have been reported. The antibodies were detected by Western blotting only when sera were used at low dilution and none were positive in standard commercial ELISA kits, which use sera at higher dilutions. Nevertheless, these results are significant since they demonstrate that the viral antigens were expressed and available to the chicken immune system. Improving the consistency of immunisation remains a major challenge.

The integration and transcription of transgenes, both for vvVP2 or gI, was shown in all the transgenic populations and for gI it was possible by Western blotting to identify proteins of the expected mass within extracts of sporulated oocysts. Antibodies against gI recognised two bands in the parasite extract and these could potentially correspond to unmodified and modified forms. Unmodified and N-glycosylated forms have been described for native viral gI [24]. The *E. tenella* proteome has been shown to include multiple proteins associated with glycosylation, including N-glycosylation [25], and while the modifications may not be identical in the two organisms, post-translational changes may explain the occurrence of two bands in transgenic, but not wild-type parasites. For vvVP2, we could not directly prove protein expression in the parasites by Western blot because the antibodies expected to react with vvVP2 failed to recognise any form of recombinant vvVP2 cloned and produced in this same study. We infer vvVP2 expression by indirect methods including visualisation of mCherry expressed in the same vector frame, detection of vvVP2 transcripts and the presence of specific antibodies in immunised chickens.

The high sera concentration necessary for detection of specific antibodies against vvVP2 or gI, may relate to the total amount of foreign antigen being exposed to the chickens immune system. The immunisations in this study were done following protocols for vaccinations against *Eimeria*, in which the maximum number of oocysts inoculated was 5000 in the last immunisation. These numbers are considerably lower than those used with other live vectors such as *Salmonella* Typhimurium, for which doses from 10^7^ to 10^9^ organisms expressing VP2 were dosed [26]. Therefore, an interesting option could be the transference of these transgenic technologies to more fecund *Eimeria* species such as *E. acervulina* or *E. mitis* [27] in order to increase the number of transgene expressing organisms per immunisation.

Further, the transgenic populations used for the immunisations were unstable with respect to transgene retention and expression. Chickens were infected with populations enriched by FACS to contain between 85 % and 95 % fluorescent oocysts, however the harvested oocyst output collected after each passage always contained less than 22 % fluorescent oocysts. Some possible reasons for this instability have been discussed previously by Clark et al. [9], including genetic segregation and recombination leading to transgene loss. Working with stable lines of transgenic parasites which maintain transgene expression after every passage might improve the efficacy of vaccination, although the difficulties associated with obtaining clonal lines precludes routine use during medium throughput testing. Specifically, *Eimeria* cannot be cloned *in vitro* by serial dilution or similar procedures. Instead, single sporocyst infection of chickens is needed to clone *Eimeria*, which is a time-consuming and inefficient process [11, 28]. Non-clonal populations that have 100 % transgenic parasites can be obtained by applying drug selection over several *in vivo* passages to parasites where a drug-resistance cassette has been incorporated in the transfection process [9]; however, inclusion of drug-resistant genes is not appropriate for populations to be administrated as live vaccines in field chicken populations.

The choice of promoters to control timing and level of expression of the downstream transgene could be important for optimising recognition of foreign antigens by the host immune system. We characterised parasite populations expressing *vvVP2* or *gI* using 5*’Et-Actin*, a constitutive promoter used previously [9, 28] or 5’*Et-TIF*, a novel promoter identified from an RNAseq database [29] that is linked to a gene transcribed ~ 10 fold higher than *Et-Actin*. However when transgene transcripts (*vvVP2* or *gI*) under the control of the two different promoters (5*’Et-Actin* or 5*’Et-TIF*) were quantified, in relation to *mCitrine* expressed under control of *5’Et-Mic1* on the same plasmid, 5*’Et-TIF* was found to promote higher transcription for *gI*, but not for *vvVP2*, suggesting that the downstream transgene could be affecting the transcription regulation.

Protection against viral infections such as IBDV and ILTV can be achieved by the induction of a high titre of neutralising antibodies as demonstrated using the VP2 antigen for IBDV [30, 31], in contrast to more complicated immune mechanisms necessary to provide protection against other pathogens (eg. *Clostridium perfringens* [32]). While we have shown that the *Eimeria* expression system can be used to stimulate host immune recognition of foreign antigens, the levels of humoral response generated are likely to be insufficient to guarantee protection against challenge [33]. Prior to conducting an *in vivo* challenge the consistency and magnitude of immune response stimulated by transgenic *Eimeria* vaccination should be improved. Options include the use of promoters capable of inducing stronger transgene expression, or addition of targeting signals for delivery to the cell surface or secretion, which could to improve the exposition of the antigen and therefore its recognition [23, 34]. Optimization of the number of parasites per vaccinating dose or transference of the technology to more fecund *Eimeria* species might also encourage stimulation of more significant immune response. Novel technologies for directed integration such as CRISPR/Cas9 [35, 36] or the generation of clonal lines could also offer some advantages, improving accurate characterization of transgenic parasites and improving uniformity.

## Conclusions

Newly generated transgenic populations of *E. tenella* are capable of expressing viral antigens vvVP2 from IBDV and gI from ILTV. Moreover, these expressed antigens were recognised by the chicken immune system as evidenced by the presence of specific antibodies in the sera, providing the first report of specific antigen recognition from viral avian pathogens expressed by transgenic *Eimeria*. Nonetheless, the low level response detected indicates a requirement of improve transgene expression before testing as a vaccine.

## Additional file

Additional file 1: Table S1. Primers used in the study. **Table S2**. Analysis of post infection serum. **Figure S1**. Western blot of total *E. tenella* sporozoite extract against chicken sera immunised by infection with different transgenic or wild-type populations of *E. tenella*. Lane 1: *Et*-Act-vvVP2-P6; lane 2: *Et*-TIF-vvVP2-P2; lane 3: *Et*-Act-gI -P5; lane 4: *Et*-TIF-gI-P2; lane 5: *E. tenella* (wt); lanes 6–7: non-immunised; lane 8: positive control (anti-EtMic2); lane 9: Coomassie blue of *E. tenella* sporozoite extract; lane 10: Mw. Rabbit anti-chicken IgG antibody HRP conjugate or goat anti-mouse IgG antibody HRP (for EtMic2) conjugate at 1/1000 dilution were used as secondary antibody. (DOCX 3094 kb)

## Abbreviations

BSA: Bovine serum albumin
CjaA: *Campylobacter jejuni* antigen
ConA: Concanavalin A
ELISA: Enzyme-linked immunosorbent assay
EtMic1: *Eimeria tenella* microneme protein 1
EYFP: Enhanced yellow fluorescent protein
FACS: Fluorescence-activated cell sorting
FBS: Foetal bovine serum
gB/I/D: Virus glycoprotein B/I/D
HRP: Horseradish peroxidase
HVT: Herpes virus of turkey
IBDV: Infectious bursal disease virus
ILTV: Infectious laryngotracheitis virus
IMAC: Immobilized metal ion affinity chromatography
MDBK: Madin-Darby bovine kidney
PBMC: Peripheral blood mononuclear cells
qPCR: Quantitative PCR
REMI: Restriction enzyme-mediated integration
RT-PCR: Real time PCR
SDS-PAGE: Sodium dodecyl sulphate polyacrylamide gel electrophoresis
TIF: Translation initiation factor
vvVP2: Very virulent viral protein 2 (capsid protein).
