# Supplementary material for: Viral proteins expressed in the protozoan parasite Eimeria tenella are detected by the chicken immune system
Source: Parasit Vectors. 2016 Aug 23;9(1):463. doi: 10.1186/s13071-016-1756-2 (PMC4994267; doi:10.1186/s13071-016-1756-2)
Supplement: Additional file 1: Table S1. — Primers used in the study. Table S2. Analysis of post infection serum. Figure S1. Western blot of total E. tenella sporozoite extract against chicken sera immunised by infection with different transgenic or wild-type populations of E. tenella. Lane 1: Et-Act-vvVP2-P6; lane 2: Et-TIF-vvVP2-P2; lane 3: Et-Act-gI -P5; lane 4: Et-TIF-gI-P2; lane 5: E. tenella (wt); lanes 6–7: non-immunised; lane 8: positive control (anti-EtMic2); lane 9: Coomassie blue of E. tenella sporozoite extract; lane 10: Mw. Rabbit anti-chicken IgG antibody HRP conjugate or goat anti-mouse IgG antibody HRP (for EtMic2) conjugate at 1/1000 dilution were used as secondary antibody. (DOCX 3094 kb) [file 13071_2016_1756_MOESM1_ESM.docx]

**Table S1.** Primers used in the study

| **Name** | **Sequence** |
| --- | --- |
| **Plasmids constructs - Set 1 (2.3)** | |
| Fw-vvVP2-XhoI | CCCTCGAGGGACAAACCTGCAAGAT |
| Rv-vvVP2-BglII | GAAGATCTTCTTACCTCCTTATAGCCCGGA |
| Fw-gI-XhoI | CCCTCGAGGGGCATCGCTACTTGGAA |
| Rv-gI-BglII | GAAGATCTTCTCACATTTTTATTGAGTCGGGC |
| Fw-Che-XbaI | GCTCTAGAATGGCGACCATGGTG |
| Rv-Che-BglII | GAAGATCTCTACTCTGCTGCAAACCTCG |
| **Plasmids constructs - Set 2 (2.3)** | |
| Fw-5’TIF-ClaI (*vvVP2*) | CCATCGATACACCCAAAACTAAAGGC |
| Fw-5’TIF-BamHI (*gI, mChe*) | CGGGATCCACACCCAAAACTAAAGG |
| Rv-5’TIF-XbaI | GCTCTAGATTGAGACATCTACACTGC |
| **PCR (gDNA, cDNA) & RT-qPCR (cDNA) (2.7, 2.8)** | |
| Fw-VP2-RT | CCCAGAGTCTACACCATAAC |
| Rv-VP2-RT | GCTGAGAACAGTGTGATTGT |
| Fw-gI-RT | CACCCGTTTAAGCTCACTAT |
| Rv-gI-RT | GGGTTCTTTGGTGTCGTCTA |
| Fw-Act-Ex | GAACTCCAAAACACTTCGCC |
| Rv-Act-Ex | CACTTGGCGCCGAGCCAGAC |
| Fw-Act-Int | TTGTTGTGGTCTTCCGTCA |
| Rv-Act-Int | GAATCCGGGGAACATAGTAG |
| **qPCR (gDNA) (2.8)** | |
| Fw-mCitrine | CTGCACCACCGGCAA |
| Rv-mCitrine | GTAGCGGGCGAAGCACT |
| Fw-5S | TCATCACCCAAAGGGATT |
| Rv-5S | TTCATACTGCGTCTAATGCAC |
| **Recombinant proteins (2.9)** | |
| Fw-pET32b-vvVP2-HindIII | CCCAAGCTTATTGTGGGTGCTCACTAC |
| Rv-pET32b-vvVP2-XhoI | CTCGAGCTGTGATGAGAATTGGTA |
| Fw-pET32b-gI-HindIII | CCCAAGCTTTTCTACGTAATTGTTCGG |
| Rv-pET32b-gI-XhoI | CTCGAGCGTAGGGCTCATTGTCCC |
| Fw-pET32b-gD-HindIII | CCCAAGCTTGCTGCGCTCTCTGGCCAA |
| Rv-pET32b-gD-XhoI | CTCGAGCGGGATCACGGCGGGCAT |

**Table S2.** Analysis of post infection serum

| Group | ELISA-GDV (S/P)^1^ | ELISA-GDV Titre (2Log) | VN-GDV Titre (2Log) |
| --- | --- | --- | --- |
| *Et-*Act-vvVP2 (G1) | -0.008 | Neg | < 5 |
|  | 0.008 | Neg | < 5 |
|  | -0.008 | Neg | < 5 |
|  | -0.014 | Neg | < 5 |
|  | 0.008 | Neg | < 5 |
|  | 0.036 | Neg | < 5 |
|  | 0.003 | Neg | < 5 |
|  | 0.014 | Neg | < 5 |
|  | 0.003 | Neg | < 5 |
|  | -0.008 | Neg | < 5 |
|  | -0.019 | Neg | < 5 |
|  | 0.157 | Neg | < 5 |
| *Et-*TIF-vvVP2 (G2) | 0.003 | Neg | < 5 |
|  | -0.014 | Neg | < 5 |
|  | -0.019 | Neg | < 5 |
|  | -0.014 | Neg | < 5 |
|  | 0.008 | Neg | < 5 |
|  | -0.014 | Neg | < 5 |
|  | -0.019 | Neg | < 5 |
|  | -0.019 | Neg | < 5 |
|  | -0.003 | Neg | < 5 |
|  | -0.003 | Neg | < 5 |
|  | -0.014 | Neg | < 5 |
|  | 0.008 | Neg | < 5 |
| *E. tenella* (wt) (G3) | -0.014 | Neg | < 5 |
|  | -0.008 | Neg | < 5 |
|  | -0.008 | Neg | < 5 |
|  | -0.014 | Neg | < 5 |
|  | -0.019 | Neg | < 5 |
|  | -0.030 | Neg | < 5 |
| Group | ILT ELISA (S/P)^2^ | ILT ELISA Titer (2Log) |  |
| *Et-*Act-gI (G4) | 0.02 | Neg |  |
|  | -0.01 | Neg |  |
|  | -0.01 | Neg |  |
|  | 0.05 | Neg |  |
|  | 0.02 | Neg |  |
|  | 0.01 | Neg |  |
|  | 0.01 | Neg |  |
|  | 0.03 | Neg |  |
|  | 0.00 | Neg |  |
|  | 0.00 | Neg |  |
|  | 0.00 | Neg |  |
|  | 0.02 | Neg |  |
| *Et-*TIF-gI (G5) | 0.01 | Neg |  |
|  | 0.01 | Neg |  |
|  | 0.01 | Neg |  |
|  | 0.01 | Neg |  |
|  | 0.02 | Neg |  |
|  | 0.01 | Neg |  |
|  | 0.04 | Neg |  |
|  | 0.01 | Neg |  |
|  | 0.01 | Neg |  |
|  | 0.00 | Neg |  |
|  | 0.01 | Neg |  |
| *E. tenella* (wt) (G6) | 0.03 | Neg |  |
|  | 0.01 | Neg |  |
|  | 0.01 | Neg |  |
|  | 0.03 | Neg |  |
|  | 0.00 | Neg |  |
|  | 0.01 | Neg |  |

^1^ Sample to Positive Ratio (S/P) ≤ 0.200 = Neg (negative)

^2^ Sample to Positive Ratio (S/P) ≤ 0.150 = Neg (negative)


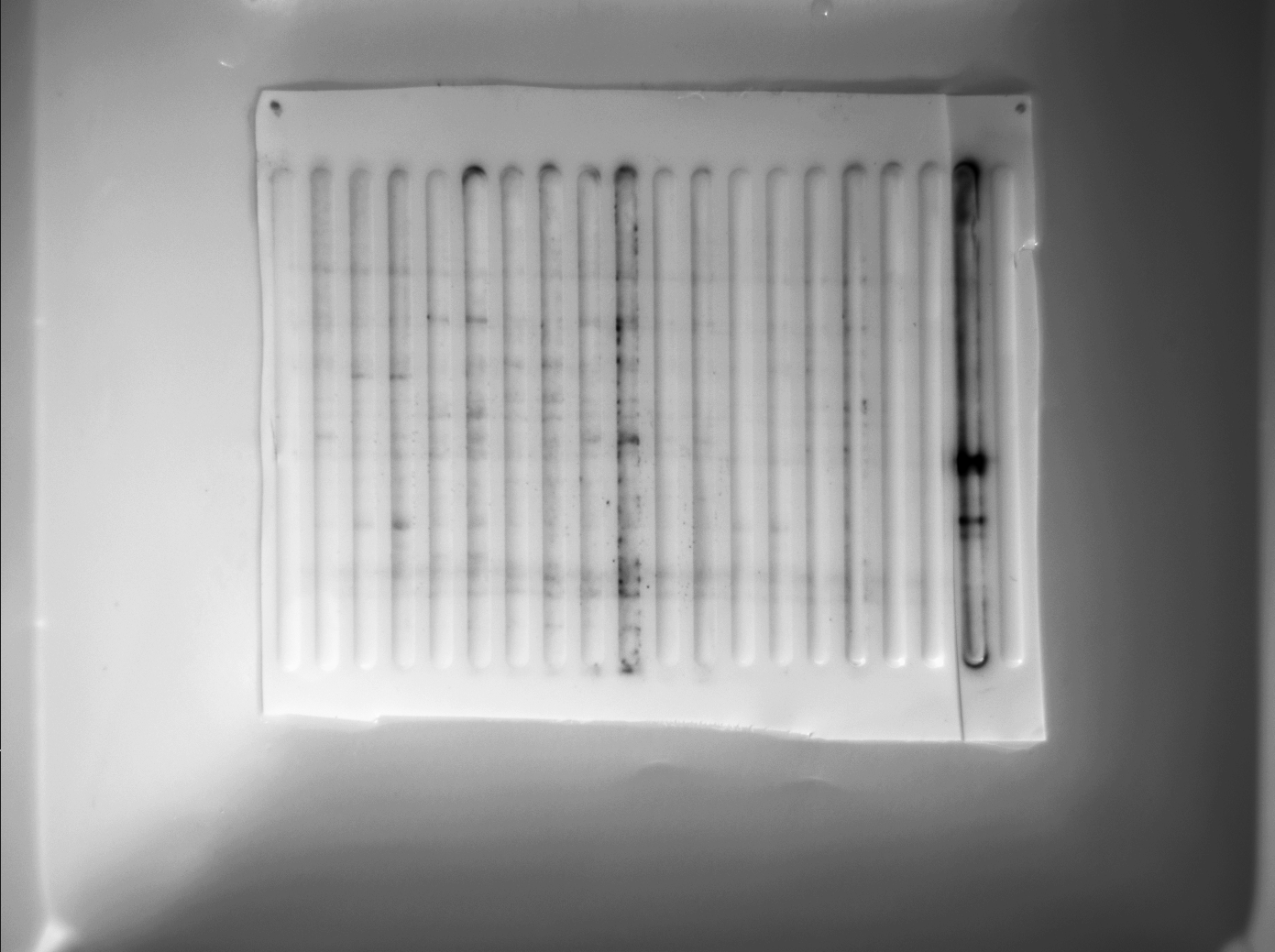

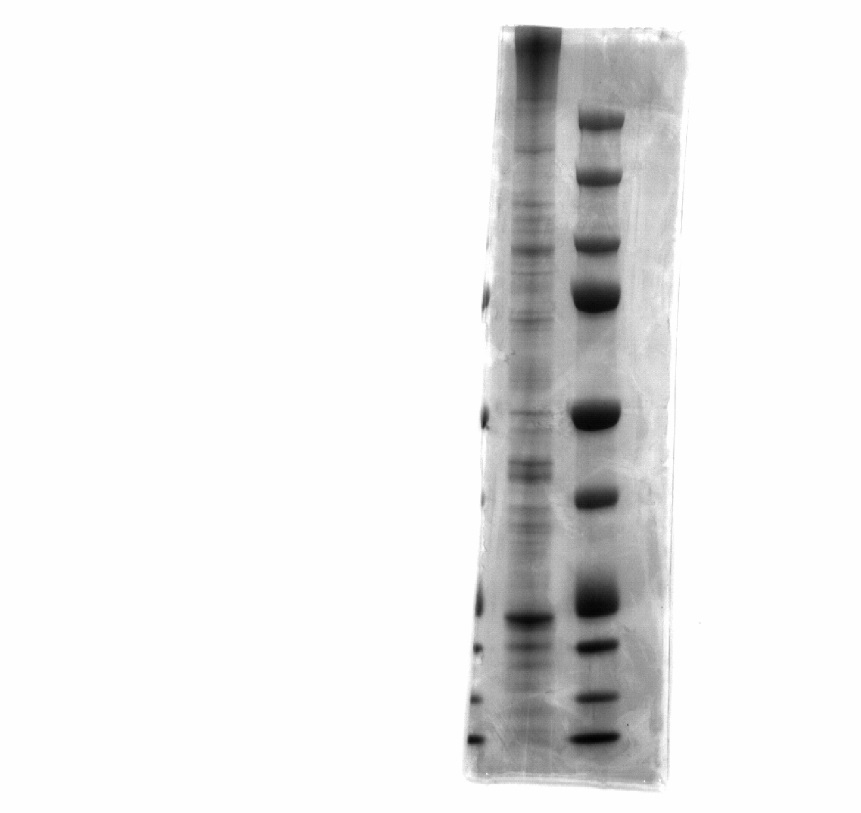


1 2 3 4 5 6 7 8 9 10

50

37

25

20

75

100

150

250

kDa

15

10

**Figure S1.** Western blot of total *E. tenella* sporozoite extract against chicken sera immunised by infection with different transgenic or wild-type populations of *E. tenella*. Lane 1: *Et*-Act-vvVP2-P6; lane 2: *Et*-TIF-vvVP2-P2; lane 3: *Et*-Act-gI -P5; lane 4: *Et*-TIF-gI-P2; lane 5: *E. tenella* (wt); lanes 6-7: non-immunised; lane 8: positive control (anti-EtMic2); lane 9: Coomassie blue of *E. tenella* sporozoite extract; lane 10: Mw. Rabbit anti-chicken IgG antibody HRP conjugate or goat anti-mouse IgG antibody HRP (for EtMic2) conjugate at 1/1000 dilution were used as secondary antibody.
